# Validity and Reliability of the Amharic Version of the World Health Organization's Quality of Life Questionnaire (WHOQOL-BREF) in Patients with Diagnosed Type 2 Diabetes in Felege Hiwot Referral Hospital, Ethiopia

**DOI:** 10.1155/2019/3513159

**Published:** 2019-05-06

**Authors:** Kidist Reba, Bizuayehu Walle Birhane, Hordofa Gutema

**Affiliations:** ^1^Department of Adult Health Nursing, School of Health Science, College of Medicine and Health Science, Bahir Dar University, P.O. Box 79, Ethiopia; ^2^Department of Physiology, School of Medicine, College of Medicine and Health Science, Bahir Dar University, P.O. Box 79, Ethiopia; ^3^Department of Health Promotion and Behavioral Sciences, School of Public Health, College of Medicine and Health Science, Bahir Dar University, P.O. Box 79, Ethiopia

## Abstract

**Background:**

Although it is largely preventable, type 2 diabetes is the most common type and accounts for the vast majority of diabetes cases worldwide.

**Objective:**

To assess the validity and reliability of the Amharic version of the abbreviated World Health Organization Quality of Life (WHOQOL-BREF) instrument for measuring quality of life in people with diagnosed type 2 diabetes.

**Methods:**

The Amharic version of the abbreviated World Health Organization Quality of Life instrument was administered to 344 patients with diagnosed type 2 diabetes in Felege Hiwot Referral Hospital. Contribution of domain scores to QOL facets was assessed using multiple linear regression. Reliability assessment was done by using Cronbach's alpha coefficient. Construct validity was examined using exploratory and confirmatory factor analyses.

**Result:**

The Amharic version of the abbreviated World Health Organization Quality of Life instrument has acceptable internal consistency. Confirmatory factor analysis has shown acceptable goodness of fit for 4 domain models. The physical, psychological, and environmental domains have a statistically significant contribution in explaining overall quality of life, while only physical and psychological domains have significant contribution in explaining the general health facet.

**Conclusion:**

The Amharic version of the abbreviated World Health Organization Quality of Life instrument is appropriate for patients with diagnosed type 2 diabetes. The overall finding of analysis implies that the Amharic version of the abbreviated World Health Organization Quality of Life instrument has internal consistency and validity to investigate quality of life among patients with diagnosed type 2 diabetes, and it can be used for studies which are going to be conducted in Ethiopia.

## 1. Introduction

Diabetes mellitus (DM) is a chronic condition that occurs when there are raised levels of glucose in the blood because the body cannot produce any or enough of the hormone insulin or use insulin effectively [1]. DM is mainly classified as type 1 diabetes, type 2 diabetes, and gestational diabetes [2]. Although it is largely preventable, type 2 diabetes is the most common type and accounts for the vast majority of diabetes cases, accounting for around 90% of all cases of diabetes [2, 3].

According to International Diabetes Foundation (IDF) estimation in 2017, globally, 8.8% or 425 million adults have diabetes. If this trend continues, 693 million of people will have diabetes by 2045, and the larger increases will occur in low- and middle-income countries. Out of this number, about 79% of them live in low- and middle-income countries, and Ethiopia is the leading country in Africa having 2.6 million of people with diabetes [2]. In addition to premature mortality and negative economic impact, an individual with diabetes will have lower quality of life (QOL) due to a diabetes-related complication [4, 5].

World Health Organization (WHO) defines quality of life as an individual's perception of their position in life in the context of the culture and value systems in which they live and in relation to their goals, expectations, standards, and concerns [6]. Although it is well known to involve poor body regulation of blood glucose, little attention is given to the impact of the diabetes-related illness on the perception that individuals have of their social relationships, working capacity, and financial status [7]. To enable researching on such situations, WHO Quality of Life (WHOQOL) instruments were developed focusing on individuals' own views of their well-being and satisfaction with their functioning and with effects of treatment [6].

WHOQOL-100 is a cross-culturally valid measure of well-being which is operationalized through 100 items of 25 facets organized in six domains: physical, psychological, levels of independence, social relationship, environment, and spirituality [8, 9]. The WHOQOL-BREF is a short version of WHOQOL-100, and it was developed in four domains of QOL: physical, psychological, social, and environmental for use in situations where time is limited, minimizing respondent burden, and considering as facet-level detail is not important [10]. The WHOQOL-BREF is initially available in 19 languages after being evaluated for cross-cultural validity in twenty field centers in 18 countries [7]. Following this, different scholars have validated and used it in a version that is appropriate to their setting [11–16].

In Ethiopia, studies were conducted using WHOQOL-BREF to measure the QOL of different population groups [17–19]. However, other than one study which has used HIV-specific WHOQOL-BREF (WHOQOL-HIV-BREF), none of these studies have validated the Amharic (official language of Ethiopia) version of WHOQOL-BREF [20]. Thus, the current study is aimed at the validation of the Amharic version of WHOQOL-BREF which is designed for measuring QOL of people with diagnosed type 2 diabetes in Felege Hiwot Referral Hospital.

## 2. Methods

### 2.1. Study Setting and Population

The study was conducted on three hundred forty-four patients with diagnosed type 2 diabetes who were recruited from the DM follow-up clinic of Felege Hiwot Referral Hospital (FHRH). FHRH is located in Bahir Dar City, which is 565 km from Addis Ababa, the capital of Ethiopia. DM patients constitute a large number of patients attending in the follow-up clinic. There are 1,678 type 2 DM patients following up in the clinic currently. Patients diagnosed with type 2 diabetes who had follow-up for ≥1 year, aged >18 years, and visited the facility for follow-up during the study period were selected and participated in this study after excluding patients who had history of substance abuse such as alcohol and cigarette. A detailed description sampling procedure is available elsewhere [21].

### 2.2. Instrument

The English version of the WHOQOL-BREF instrument was translated to Amharic according to the guidelines developed by the WHOQOL Group [6]. The WHOQOL-BREF is a 26-item instrument consisting of four domains: physical health (7 items), psychological health (6 items), social relationships (3 items), and environmental health (8 items), and two items not included in any of the domains are overall perception of QOL and general health. Each of these items were scored from 1 to 5 on a response scale, which is agreed as a five-point Likert scale. The domain scores were calculated by (1) reverse coding the scores of two items of the physical domain and one item for the psychological domain which were negatively worded initially, (2) using the mean score of items within each domain to calculate the domain score, (3) multiplying the mean scores by 4 in order to make domain scores, (4) transforming the domain scores linearly to a 0-100 scale.

### 2.3. Translation of the Instrument

The English version of the WHOQOL-BREF instrument was translated into Amharic by one nurse. The translator was provided a clear and detailed information about the instrument and the population who will use the instrument to make sure that the original concept underlying each item was maintained in the translation. Then, review of the translated Amharic version was done by the panel of three bilingual speakers and writer of both English and Amharic languages. This group looked for any inconsistencies between the source language version and the translated document and resolved issues related to the maintenance of the integrity of the source instrument. The Amharic version of the instrument was then translated back to English by one translator who is a lecturer at the Department of English Language and Literature, Bahir Dar University, and independent of the first translator. A meeting of investigators and translators was held to discuss and approve the final version of the questionnaire.

### 2.4. Statistical Analysis

Data analyses were performed using SPSS 20.0 for Windows. Descriptive statistics like mean and standard deviation and percentage for continuous variables were done. Reliability assessment was done by checking internal consistency using Cronbach's alpha coefficient. The alpha coefficient with 0.70 and above was accepted as evidence of internal consistency of each domain of WHOQOL-BREF. Contribution of domain scores to QOL facets was assessed using multiple linear regression. Construct validity was examined using exploratory factor analysis (EFA) and confirmatory factor analysis (CFA).

EFA was conducted by Principal Component Analysis (PCA) with varimax rotation. The Kaiser-Meyer-Olkin (KMO) value greater than 0.5 was used as the adequacy of the sample size for EFA. Eigenvalue was used to decide the number of factors to retain; those factors with Eigenvalue greater than or equal to 1 were retained. To determine the number of items loaded on each factor, factor-item correlation of 0.30 and above was set for inclusion in factor loading.

CFA was done using EQS version 6.3 to evaluate which model best fits for the four-model structure yielded by EFA. In this analysis, relative chi-square (*χ*^2^/df), the comparative fit index (CFI), a nonnormed fit index (NFI), and the root mean square error of approximation (RMSEA) were used as the model fit indices. Acceptable threshold levels for fit index CFI, NNFI, and RMSEA were >0.90, >0.95, and <0.7, respectively [22].

## 3. Result

### 3.1. HRQOL Scores of the Study Participants

The mean score of WHOQOL-BREF domains is presented in Table 1. Among the four domains of WHOQOL-BREF of type 2 diabetic patients, the social health domain has a higher mean score (57.8 ± 14.8 SD), while the physical domain presents with a lower mean score (48.1 ± 20.4 SD) (Table 1).

### 3.2. Reliability

Reliability analysis has showed Cronbach's alpha value of above 0.7 for the domains of HRQOL except for the social domain which is 0.58. Among the four domains, the physical domain has the highest (0.84) alpha coefficient (Table 1).

### 3.3. Construct Validity

The KMO measure of sample size adequacy was checked prior to factor analysis. The KMO measure of sample size adequacy was 0.849 showing its appropriateness for factor analysis [23]. After reversely coding negatively worded items, four-factor model EFA was carried out on 24 items using the principal component analysis with the varimax rotation factor extraction method to extract the 4 domains of original WHOQOL-BREF, and this model explained 47.54% of the total variance. In this model, item Q22 (satisfaction with support from friend) was dropped for having low factor loading (<0.3). Except for factor 3, which retained only half of the original psychological domain, a majority of the items loaded in the other 3 factors were similar to the original WHOQOL-BREF domains.

Factor 1 included all of the 7 items of the physical domain and 3 items (life enjoyment, self-satisfaction, and negative feeling) of the original psychological domain, and it explained 22.62% of the variance. Factor 2 consisted 7 items of the original environmental domain, and it explained 10.53% of the variance. Factor 3 has retained 3 items (feeling meaningful life, concentrate on daily activity, and accepting bodily appearance) of the original psychological domain and safe daily life from the environmental domain, and this factor explained 7.35% of the variance. Two items of the original social health domain and an item of physical environment of the original environmental domain were included in factor 4, and it explained 7.03% of the variance (Table 2).

The original model of CFA has shown CFI = 0.86, NNIF = 0.84, and RMSEA = 0.07 (90% CI: 0. 068, 0.081) with chi − square/df = 706/244. However, goodness of fit indices become CFI = 0.94, NNFI = 0.95, and RMSEA = 0.055 (0.044 - 0.066) with chi − square/df = 194/95, after removing 2 items (concentrate on daily activity of the psychological domain and satisfaction with living place of the environmental domain) for showing the *r*^2^ < 0.20 during the assessment of goodness of fit for individual domains [22] and when item accepting bodily appearance from the original psychological domain and physical environment from the original environmental domain were allowed to cross-load on physical health and social domains, respectively (Table 3).

### 3.4. Association between Domains and Overall Quality of Life

Multiple linear regression was carried out to determine the contribution of each WHOQOL-BREF domain score on facets of quality of life and to explain the observed variance of the models. As presented in Table 4, except the social relationship domain, all the other three domains are significantly associated with the overall quality of life facet, and only physical and psychological domains were significantly associated with general health facets. The physical health and psychological domains have a higher statistically significant contribution on overall quality of life and general health, respectively. The two models have explained 48% (*R*^2^ = 0.48) and 42% (*R*^2^ = 0.42) of observed variance for overall QOL and general health, respectively (Table 4).

## 4. Discussion

The aim of the current study was to assess the validity and reliability of the Amharic version of the WHOQOL-BREF in patients with diagnosed type 2 diabetes. Crombach's alpha coefficients for the physical health domain, psychological domain, social health domain, and environmental health were 0.84, 0.74, 0.58, and 0.71, respectively. Except for the social health domain, Crombach's alpha coefficients of the physical health domain, psychological domain, and environmental health domain have shown Crombach's alpha coefficients of the above acceptable value 0.7 [24], indicating good internal consistency of this tool. A lower alpha coefficient of the social health domain was also reported in other studies. In a study conducted in Taiwan among patients with pulmonary tuberculosis, Crombach's alpha coefficient of the social health domain was 0.61 [12]. A pilot study conducted in field centers of the WHOQOL Group to develop and test the WHOQOL-BREF instrument has also reported 0.66 Crombach's alpha value for the social health domain [6]. In a study conducted to develop Korean versions of the WHO Quality of Life scale and WHOQOL-BREF, Crombach's alpha value for the social health domain was 0.58 [25]. Failing to use the recommended minimum of four items in assessment of internal consistency in this domain may be the reason for its lower alpha coefficient.

Regarding the validity, factor analysis has shown an acceptable finding. In EFA, factors 1 and 2 have consisted of similar items with the original physical and environmental domains, respectively. Five items are loaded on another domain different from their original one. Among these items, items life enjoyment, self-satisfaction, and negative feeling of the original psychological domain were loaded on the physical domain, while items safe daily life and physical environment of the original environmental domain were loaded on the psychological domain and social domain, respectively. It is also important to note that the item accepting bodily appearance from the original psychological domain and physical environment from the original environmental domain had cross-loaded on physical health and social domains, respectively. Cross-loading of physical and psychological domain items was also reported in a study which validated WHOQOL-BREF for patients with type 2 DM in India [16]. Although it was done among people with physical disability, a study conducted in Korea to validate WHOQOL-BREF revealed cross-loading of environmental domain items with the social health domain [14]. In a study conducted in Taiwan to investigate the quality of life of traumatic spinal cord injury using WHOQOL-BREF, cross-loading of ten items with other domains was observed [26].

In the CFA, the original model has shown indices of CFI = 0.86, NNIF = 0.84, and RMSEA = 0.07, indicating that the domains in the model did not fit for the patients diagnosed with type 2 diabetes. However, the model gained acceptable goodness of fit (CFI = 0.94, NNIF = 0.95, and RMSEA = 0.05), after removing 2 items (concentrate on daily activity of the psychological domain and satisfaction with living place of the environmental domain) for showing *r*^2^ < 0.20 during the assessment of goodness of fit for individual domains and when item accepting bodily appearance from the original psychological domain and physical environment from the original environmental domain were allowed to cross-load on physical health and social domains, respectively. This is in line with the findings of different studies which used WHOQOL-BREF. In a validation study conducted among substance users in northern Taiwan, the modified model has shown acceptable goodness of fit of indices CFI = 0.92, NNFI = 0.91, and RMSEA = 0.06 [11]. In a study conducted among older people in Taiwan, the CFI was increased to 0.90 from 0.85 when three pairs of error variances were allowed to covary and two items were allowed to cross-load on other domains [27]. A study which was conducted in Netherlands among women with breast cancer has found improvement in model fit (CFI = 0.90; RMSEA = 0.06) when error variances were allowed to covary [28]. However, Skevington et al. study which was conducted among adults in 23 countries and Jaracz et al. Polish study has reported CFI value of 0.863 and 0.87, respectively [13, 15].

The current study has also confirmed that the physical domain has highly contributed for the overall quality of life, while the psychological domain is the main contributor of general health, indicating that physical and psychological domains are strong contributors of quality of life. The finding of other studies was different from the current one. In a study of Korean people with physical impairments, the psychological domain was the main contributor of overall quality of life, whereas the physical domain is highly associated with general health [14]. A study of Polish respondents has also showed that the psychological domain has the strongest contribution for overall quality of life, whereas the physical domain was the contributor of general health followed by the psychological domain [13]. However, the physical domain was found to be a strong contributor for both overall quality of life and general health in a study conducted in India among people with type 2 diabetes [16]. The social domain has no significant contribution in both quality of life facets in the current study. In a study of Korean people, the social domain has no contribution in both overall quality of life and general health [14]. The social domain has only association with overall quality of life in a study conducted in Polish respondents [13].

This study has some limitations. First, a cross-sectional study design was used, and it lacks reporting of causal relationship of the variables. Second, since the data was collected using an interviewer-administered questionnaire, the finding of this study might be prone to social desirability bias, and psychometric properties of some of the items might be affected. Third, assessment of reliability of the instrument was done only by testing of internal consistency, and test-retest reliability was not examined.

## 5. Conclusion

The finding of this study revealed that the Amharic version of the WHOQOL-BREF has acceptable internal consistency and construct validity for investigating QOL in patients with diagnosed type 2 diabetes, and it can be used for studies going to be conducted in Ethiopia. Physical and psychological domains are found to be strong contributors of overall quality of life and general health. The social health domain has lower internal consistency, and it has no contribution for the quality of life; thus, further exploration is needed to improve the reliability of the social health domain of WHOQOL-BREF.

## Figures and Tables

**Table 1:** Internal consistency and mean score of WHOQOL-BREF domains in patients with diagnosed type 2 diabetes attending FHRH (*n* = 344).

Domains of HRQOL	*N* of items	Mean ± SD	Min score (%)	Max score (%)	Alpha
Physical health domain	7	48.1 ± 20.4	7.1	92.9	0.84
Psychological domain	6	52.1 ± 16.3	4.2	87.5	0.74
Social health domain	3	57.8 ± 14.8	0.00	91.7	0.58
Environmental domain	8	52.3 ± 13.0	6.3	84.4	0.71

**Table 2:** Exploratory factor analysis of the Amharic version of WHOQOL-BREF in patients with diagnosed type 2 diabetes attending FHRH (*n* = 344).

Items	Item description	Factor 1	Factor 2	Factor 3	Factor 4
Q3	Pain	0.757			
Q4	Medical dependency	0.735			
Q10	Enough energy for everyday life	0.632			
Q15	Ability to get around	0.587			
Q16	Satisfaction with sleep	0.742			
Q17	Satisfaction with daily living activity	0.653			
Q18	Satisfaction with capacity for work	0.688			
Q5	Life enjoyment	0.551			
Q6	Feeling meaningful life			0.433	
Q7	Concentrate on daily activity			0.648	
Q11	Accepting bodily appearance	0.428		0.498	
Q19	Self-satisfaction	0.723			
Q26	Negative feeling	0.660			
Q20	Satisfied with personal relationships				0.526
Q21	Satisfaction with sex life				0.468
Q8	Safe daily life			0.568	
Q9	Physical environment		0.412		0.582
Q12	Enough money to meet the needs		0.670		
Q13	Availability of daily information		0.688		
Q14	Opportunity for leisure activities		0.570		
Q23	Satisfaction with living place		0.520		
Q24	Satisfied with access to health services		0.462		
Q25	Satisfaction with mode of transportation		0.653		
Variance explained (%)		22.62	10.53	7.35	7.04
Total variance explained (%)		47.54			

**Table 3:** Goodness of fit indices for confirmatory factor analysis of the Amharic version of WHOQOL-BREF in patients with diagnosed type 2 diabetes attending FHRH (*n* = 344).

	Suggestion	*χ* ^2^/df	CFI	NNIF	RMSEA (90% CI)
Original model		706/244	0.86	0.84	0.07 (0.068-0.081)
Modified model	Remove (Q7 & Q23)	338.6/128	0.89	0.88	0.069 (0.06 -0.078)
Final model	Add Soc-En9	194/95	0.94	0.95	0.055 (0.044 - 0.066)

*χ* ^2^: chi-square; df: degree of freedom; CFI: comparative fit index; NNFI: nonnormed fit index; CI: confidence interval; RMSEA: root mean square error of approximation.

**Table 4:** Association of the domains with overall quality of life and general health facets of the Amharic version of WHOQOL-BREF in patients with diagnosed type 2 diabetes attending FHRH (*n* = 344).

Domains	Overall QOL		General health	
	Beta	*P* value	Beta	*P* value
Physical domain	0.387	0.000	0.256	0.000
Psychological domain	0.195	0.001	0.400	0.000
Social domain	-0.038	0.368	0.052	0.221
Environmental new	0.261	0.000	0.083	0.067
Explained variance	*R* ^2^ = 0.48		*R* ^2^ = 0.42	

## Data Availability

The data used to support the findings of this study are available from the corresponding author upon request.
